# Clinical Outcomes of an Individualized Multimodal Chronic Wound Management Protocol Using Type I Collagen–Hyaluronic Acid Pads: Retrospective Observational Study

**DOI:** 10.3390/jpm16080412

**Published:** 2026-07-31

**Authors:** Cristina Stanescu, Catalina Teodora Pintilie, Roxana Elena Bogdan Goroftei, Madalina Nicoleta Matei, Iulia Chiscop, Monica Boev, Florina Popa, Camelia Tamas

**Affiliations:** 1 “Dunărea de Jos” University of Galați, Faculty of Medicine and Pharmacy, Department of Morphological and Functional Sciences, Medical-Pharmaceutical Research Center, Integrated Laboratory of Microbiology, Immunology, Nutrition, and Genetics, Domnească Street no. 47, 800008 Galati, Romania; 2 “Grigore T. Popa” University of Medicine and Pharmacy, Faculty of Medicine, Department ENT, 16 Universitatii Street, 700115 Iasi, Romania; 3 “Dunărea de Jos” University of Galați, Faculty of Medicine and Pharmacy, Department of Morphological and Functional Sciences, Domnească Street no. 47, 800008 Galati, Romania; 4 “Dunărea de Jos” University of Galați, Faculty of Medicine and Pharmacy, Department of Dental Medicine, Domnească Street no. 47, 800008 Galati, Romania; 5 “Dunărea de Jos” University of Galați, Faculty of Medicine and Pharmacy, Clinical Surgical Department, Domnească Street no. 47, 800008 Galati, Romania; 6 “Dunărea de Jos” University of Galați, Faculty of Medicine and Pharmacy, Department of Pharmaceutical Sciences, Domnească Street no. 47, 800008 Galati, Romania; monica.boev@ugal.ro; 7 “Dunărea de Jos” University of Galați, Faculty of Medicine and Pharmacy, Clinical Surgical Department, Medical-Pharmaceutical Research Center, Integrated Laboratory of Surgical Techniques and Medical Imaging, Domnească Street no. 47, 800008 Galati, Romania; florinapopa11@yahoo.com; 8 “Grigore T. Popa” University of Medicine and Pharmacy, Faculty of Medicine, Clinical Surgical Department, 16 Universitatii Street, 700115 Iasi, Romania

**Keywords:** wound healing rate, chronic wound, collagen–hyaluronic acid pads, bacterial colonization

## Abstract

**Background**: Chronic wounds pose a persistent therapeutic challenge. Type I collagen and hyaluronic acid (HA) support tissue repair, but real-world evidence for describing their combined use in padformulations is limited. This study provides a real-world description of clinical outcomes observed within a tailored management, including Type I Collagen–Hyaluronic Acid pads (HCHA), on healing trajectories across diverse chronic wound etiologies. **Methods**: This retrospective observational analysis included 64 patients treated between 2017 and 2024 for chronic wounds, including burns (*n* = 32) and trophic ulcers of various etiologies (*n* = 32). Clinical data, including wound characteristics, daily healing rate (DHR), and procedural pain assessed using repeated VAS measurements, were extracted from medical records. **Results**: The daily healing rate (DHR) ranged from 3.22% in neglected burn wounds to 2.03% in non-burn chronic wounds. Percent area reduction (PAR) analysis at 7, 14, and 30 days showed greater wound area reduction in burn wounds (20%, 36%, and 62%) compared with non-burn wounds (13%, 25%, and 45%). Procedural pain decreased in both cohorts, with burn injuries presenting higher baseline VAS scores but following a similar downward trajectory over time (*p* < 0.001), converging to comparably low levels by day 30. Overall, the findings describe the outcomes observed within an individualized, multimodal wound care approach, which incorporates HCHA pads tailored to wound-specific characteristics. **Conclusions**: In this retrospective observational study, patients treated with HCHA pads as part of individualized multimodal wound care exhibited progressive granulation, favorable wound-healing trajectories, and lower procedural pain levels. Further controlled studies are warranted to validate these results and refine the patient selection criteria.

## 1. Introduction

### 1.1. Study Objectives

The primary objective of this retrospective clinical study was to describe the effectiveness of HCHA pads in managing chronic wounds of various etiologies. This study focused on measurable clinical outcomes, including wound surface reduction, bacteriological status, granulation, and epithelialization dynamics. The secondary objectives included evaluating patient-reported procedural pain and the clinical feasibility of integrating this therapeutic approach into routine wound care. Despite numerous studies on isolated wound care interventions, there is limited clinical evidence specifically addressing how HCHA pads perform when integrated into individualized, multimodal strategies for different chronic wound etiologies. This gap is critical because real-world clinical practice often involves such complex, tailored approaches, making it essential to understand the combined efficacy and patient outcomes. This study offers a real-world description of the clinical outcomes associated with this tailored therapeutic approach, providing preliminary insights into a complex and heterogeneous area of clinical practice.

### 1.2. Role of Hyaluronic Acid in Wound Healing

HA is a key glycosaminoglycan in the extracellular matrix that is essential for maintaining tissue hydration, regulating inflammation, and facilitating cell migration. In acute wound healing, HA orchestrates keratinocyte and fibroblast activity, modulates cytokine gradients, and supports angiogenesis through interactions with CD44 and RHAMM receptors. In chronic wounds, HA levels are reduced or structurally altered, impairing its ability to bind to growth factors and stabilize the ECM. Modified forms of HA, such as sulfated HA, exhibit enhanced bioactivity, including greater growth factor affinity, improved macrophage polarization toward the M2 phenotype, and increased neovascularization. Beyond its structural and biochemical roles, HA exerts direct immunomodulatory effects that are highly relevant in chronic wound physiology. High-molecular-weight HA suppresses pro-inflammatory signaling by inhibiting NF-κB activation and reducing the expression of TNF-α, IL-1β, and IL-6. These properties make HA–based matrices particularly valuable in chronic wound management, where restoring hydration, reducing inflammation, and promoting cellular infiltration are critical therapeutic goals [1,2].

### 1.3. Importance of Type I Collagen in Extracellular Matrix Restoration

Type I collagen is the predominant structural protein of the dermis and plays a central role in restoring ECM integrity in chronic wound healing. Collagen scaffolds provide a three-dimensional framework that supports fibroblast migration, granulation tissue formation, and the deposition of new matrix components. Intact collagen fibers bind and sequester pro-inflammatory cytokines such as TNF-α and IL-1β, reducing their bioavailability and dampening excessive inflammatory signaling. Collagen fragments generated by protease activity can further influence immune responses by interacting with integrins (e.g., α2β1) and triggering downstream pathways that regulate macrophage behavior. In chronic wounds, excessive protease activity rapidly degrades endogenous collagen, leading to mechanical instability and impaired tissue regeneration. Exogenous collagen dressings counteract this imbalance by serving as sacrificial substrates for proteases, thereby reducing their destructive effects on the native ECM. Moreover, collagen matrices bind and protect growth factors, enhance hemostasis, and promote the transition from inflammation to proliferation. When combined with HA, collagen scaffolds offer synergistic benefits by coupling structural support with the biochemical modulation of the wound microenvironment [3,4].

### 1.4. Chronic Wound Management

Chronic wounds are common in clinical practice, and their management is often complex, prolonged, and resource-intensive, frequently requiring multiple surgical interventions and extended hospitalizations [5]. The healing trajectory is influenced by systemic and local factors, including comorbidities, nutritional and metabolic imbalances, anatomical location, wound depth, exposure to critical structures, and the characteristics of the microbial flora [6,7].

Chronic wound patients often have major systemic comorbidities or organ dysfunction, which exacerbate healing impairment and complicate wound evolution. Systemic parameters, such as plasma protein levels and hemoglobin concentration, play a decisive role in assessing a wound’s healing capacity. In patients with diabetes, metabolic control directly influences wound appearance and progression, and these variables must be considered when selecting reconstructive strategies for soft tissue defects [8]. Local factors, including prior trauma or surgery, radiotherapy-induced vascular damage, and chronic vascular diseases such as obstructive arteriopathy or chronic venous insufficiency, are frequently responsible for delayed or unfavorable healing outcomes [9].

Bacterial colonization or infection is another critical determinant of wound evolution, guiding therapeutic decision-making [10]. To address bacterial contamination, numerous studies have evaluated the effectiveness of various dressings and topical agents for wound cleansing, biofilm removal, bacterial neutralization, and prevention of antibiotic-resistant strains [11,12]. The use of topical antibiotics remains controversial due to concerns about efficacy, local adverse reactions, and the potential to promote antimicrobial resistance [13,14,15]. Recent research has highlighted the advantages of antimicrobial peptides, which exert bactericidal effects by penetrating bacterial cell membranes [16,17].

To ensure effective protection and adequate tissue hydration, modern smart dressings have been introduced, providing dynamic responses to wound conditions and supporting optimal healing trajectories. These materials conform closely to the wound surface, enhance the mobility of the affected area, and reduce the need for analgesics. Examples include multilayer dressings incorporating nano-cellulose derived from bacterial biotechnology, which provide rapid cooling and alleviate local pain, as well as advanced dressings designed to permit controlled fluid exchange between the wound and external environment [18,19].

Non-surgical wound management, including topical agents containing hyaluronic acid and collagenase, can accelerate wound cleansing, prevent wound deepening, and reduce prolonged exposure to bacterial colonization and infections [20,21,22]. Intelligent hydro-active dressings (based on hydrogels) with antibacterial activity have been developed, particularly for the wet cleaning of bacterially colonized or infected wounds. These dressings can remove bacterial biofilms from the wound surface through physical means without increasing the risk of antibiotic resistance [23]. Another advantage of intelligent dressings is that they can be left on the wound surface for several days (some for 5–7 days), which significantly reduces the risk of bacterial contamination from repeated handling or environmental exposure during dressing changes, as well as the daily need for analgesics [24,25].

**Figure 1:**
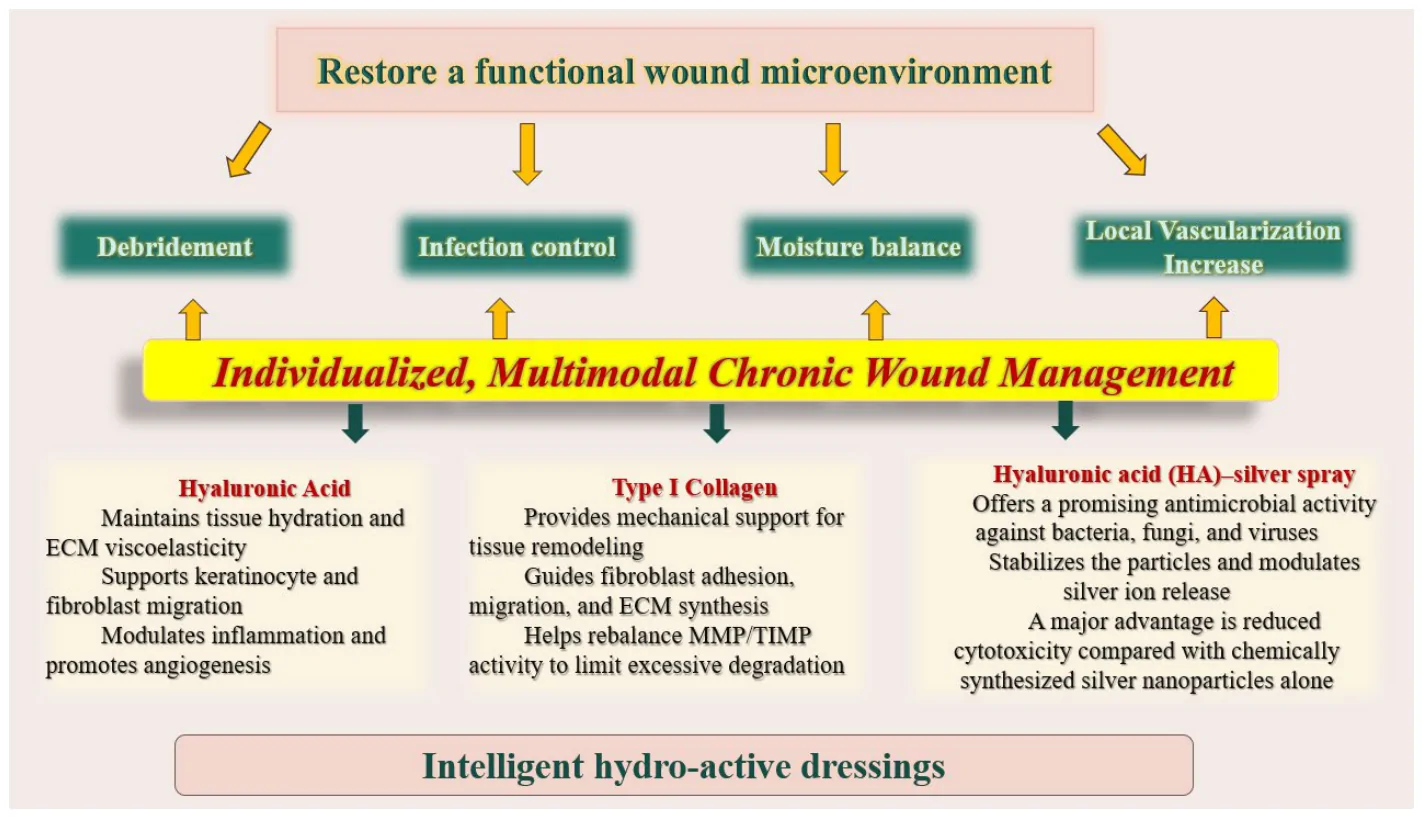
Schematic representation of key therapeutic components for establishing a functional wound microenvironment in chronic wound management, including effective debridement, infection control, maintenance of moisture balance, and stimulation of local vascularization to promote wound closure.

Chronic wound management requires a multimodal approach that addresses underlying biological dysfunctions, including persistent inflammation, impaired ECM remodeling, bacterial contamination, and tissue hypoxia [26]. Standard care encompasses surgical or enzymatic debridement, targeted infection control, maintenance of an optimal moisture environment, delivery of bioactive agents to support cellular activity, and stimulation of vascularization to facilitate wound closure (Figure 1).

Building upon this understanding, we investigated the use of Type I collagen–hyaluronic acid pads within individualized multimodal wound care, focusing on wound area reduction, healing progression, procedural comfort, and applicability across diverse chronic wound etiologies.

## 2. Materials and Methods

### 2.1. Study Population

Between 2017 and 2024, 64 patients (31 female and 33 male) with chronic wounds of diverse etiologies were evaluated and treated at the Department of Plastic Surgery, “Sf. Spiridon” Emergency Clinical Hospital in Iași, Romania. This cohort comprised 32 patients with burn injuries. The burn depth ranged from grade 2B to grade 3, with lesions located on the upper limbs (7 cases), lower limbs (8 cases), trunk (6 cases), and combined limb–trunk regions (11 cases). Patients with chronic post-burn wounds frequently present with comorbidities that could influence healing dynamics and overall clinical evolution. In this cohort, baseline conditions included anemia (4 patients), diabetes mellitus (3 patients), neoplastic diseases (4 patients), autoimmune thyroiditis (1 patient), arterial hypertension (7 patients), renal failure (4 patients), and chronic liver disease (5 patients). These comorbidities were documented at admission and deemed clinically relevant given their potential impact on tissue perfusion, immune function, metabolic balance, and susceptibility to infection.

Of the remaining 32 patients, all had non-burn chronic wounds. Within this cohort, 14 patients experienced complicated postoperative wounds with delayed healing. The wounds were located on the scalp (*n* = 2), posterior trunk *(n* = 3), anterior abdominal wall (*n* = 2), sacral region (*n* = 2), upper limb *(n* = 1), and lower limb (*n* = 4). Additionally, 18 patients presented with trophic lesions, including venous ulcers (*n* = 5), arterial ulcers (*n* = 2), pressure sores (*n* = 4), post-radiation Marjolin ulcers (*n* = 2), and diabetic foot ulcers (*n* = 5). In addition to demographic and etiological characteristics, several clinical variables were systematically assessed in all patients, including the status of bacterial contamination, wound healing rate, and pain intensity. These parameters were monitored throughout the treatment period to evaluate the clinical response and effectiveness of the therapeutic approach.

### 2.2. Inclusion and Exclusion Criteria

#### 2.2.1. Inclusion Criteria

Patients were eligible for inclusion if they met the following criteria:

- Adults (≥18 years old) diagnosed with chronic wounds with more than 4 weeks of evolution
- Chronic wounds of the following etiologies:
  - Neglected burns (grade 2B–3, 7–10% TBSA.
  - Complicated postoperative wounds.
  - Trophic ulcers (venous, arterial, pressure sores, diabetic foot ulcers).
  - Post-radiation ulcers or Marjolin ulcers (included after oncologic clearance).
- Presence of a wound requiring advanced local therapy (e.g., debridement, hydro-active dressings, collagen–hyaluronic acid pads).
- Participants must have the ability to undergo serial clinical and bacteriological monitoring.
- Patient agreement to participate in the treatment protocol and follow-up schedule.

#### 2.2.2. Exclusion Criteria

Patients were excluded if any of the following conditions were present:

- Acute wounds.
- Wounds with extensive necrosis requiring immediate major surgical excision or amputation.
- Critical limb ischemia without possibility of revascularization.
- Known allergy or intolerance to collagen, hyaluronic acid, silver-based products, or components of the applied dressings.
- Patients unable to comply with follow-up visits or treatment instructions.
- Malignant wounds requiring oncologic treatment as the primary priority.
- Pregnancy or breastfeeding (for safety considerations regarding certain topical agents).

### 2.3. Clinical Assessment and Monitoring

This investigation focused on key clinical parameters, including bacterial contamination, wound-healing rate, granulation quality, and patient-reported pain, to determine whether this therapeutic approach offers measurable advantages over standard wound-care practices.

All patients underwent serial bacteriological assessments of their wound secretions. Samples were collected at admission, during surgical debridement, and, when indicated, before soft tissue reconstruction. The diagnosis of wound infection and the role of routine bacteriological sampling remain controversial. According to the Infectious Diseases Society of America (IDSA), bacteriological sampling should be guided by clinical suspicion rather than routine [27]. Therefore, samples were collected only when at least one clinical criterion of infection was present, such as

- Clinical signs of infection;
- Wound enlargement in surface or depth despite appropriate local care;
- Formation of purulent pockets;
- Presence of purulent exudate;
- Friable granulation tissue;
- Foul-smelling secretions;
- Newly developed pain associated with local inflammation;
- Stagnation or deterioration of the wound despite correct treatment.

**Figure 2:**
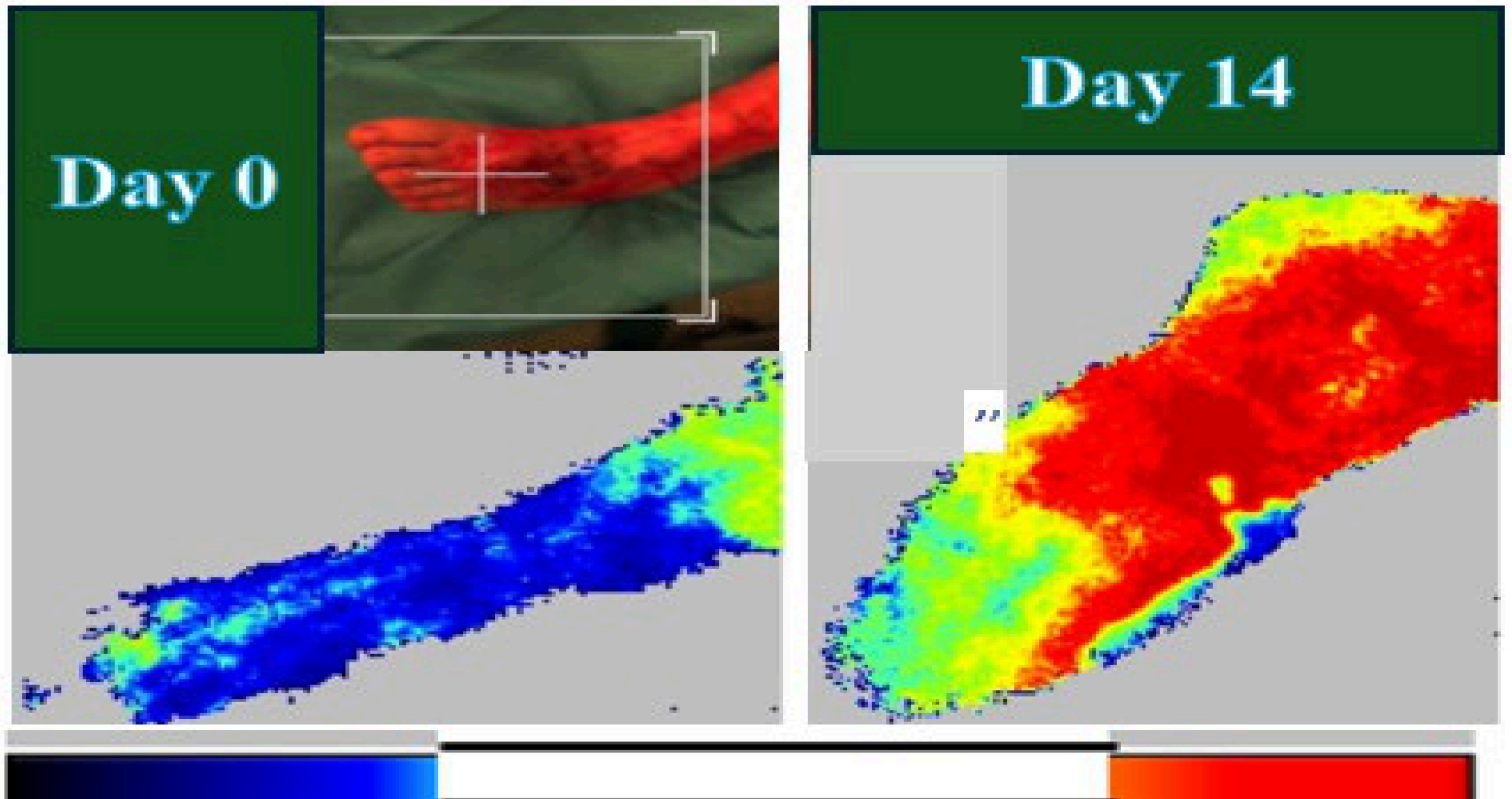
PERISCAN laser Doppler images were used to monitor local vascularization. Evolution of local vascularization between days 0 and 14. The (**left** panel) shows the initial clinical appearance of the affected foot and the corresponding perfusion map on day 0. The (**right** panel) illustrates the Day 14 perfusion image, highlighting the changes in the distribution of vascular activity. The color scale represents the relative perfusion intensity, from low (blue–green) to high (yellow–red).

Granulation tissue development and epithelialization rates were monitored in all cases. The wound surface area was calculated by dividing the lesion into geometric shapes (squares, triangles, and rectangles) and measuring their dimensions. PERISCAN Laser Doppler imaging (Figure 2) demonstrated increased local perfusion, with an estimated 100–120% improvement from day 0 to day 14, consistent with enhanced microvascular activation during the early healing phase in burn wounds.

The daily healing rate (DHR) was calculated as the percentage reduction in the initial wound area per day using the following formula:

DHR = (Initial wound area − Final wound area)/(Initial wound area × Number of days) × 100.

The wound-healing trajectory was monitored using a standardized assessment of epithelialization rate at predefined intervals. Baseline measurements were obtained on day 0, followed by sequential evaluations on days 7, 14, 21, and 30. All patients were monitored beyond the 30-day window until complete wound closure was achieved. This temporal framework enabled quantitative characterization of epithelial progression, identification of early trends in tissue regeneration, comparison of healing dynamics across treatment modalities, and detection of potential delays or deviations from the expected reparative pattern. The repeated-measures design ensured methodological uniformity and generated a reliable dataset for evaluating the dynamics of epithelial resurfacing in chronic wounds. Pain intensity during wound cleansing and dressing changes was assessed using a Visual Analog Scale (VAS) [28]. Patients with comorbidities received appropriate medical management, and imaging studies (ultrasound, Doppler, CT, and MRI) were performed when necessary. In cases of hypertrophic granulation or chronic postoperative wounds after tumor excision, tissue samples were examined microscopically to exclude recurrence [29].

### 2.4. Wound Dressings and Topical Agents

All patients received individualized wound care using dressings and topical agents, selected based on the exudate level and wound bed characteristics. HCHA pads were applied after wound bed preparation to provide structural support, maintain moisture, and promote granulation. For wounds with moderate to heavy exudates, hydro-active dressings containing superabsorbent polymers were used to ensure effective fluid management. In cases requiring enhanced exudate control and protection, multilayer high-absorption dressings with hydrophobic barrier layers were applied. When a bacterial burden was suspected, hyaluronic acid–silver spray powder was used as an antimicrobial adjunct. Conservative enzymatic debridement with collagenase was performed to remove devitalized tissue and optimize the wound bed prior to dressing application.

#### 2.4.1. Indications for Repeated Surgical or Enzymatic Debridement

Repeated debridement was performed when clinically indicated by the presence of devitalized tissue, persistent slough, fibrinous deposits, or impaired granulation. Surgical or enzymatic debridement was applied whenever these findings were judged to impede wound progression or compromise the effectiveness of subsequent interventions.

#### 2.4.2. Triggers for Antimicrobial Dressings

Antimicrobial dressings were selected based on clinical or microbiological signs suggestive of increased bioburden, including malodor, increased or purulent exudate, delayed granulation, or positive microbiological cultures. Their use was targeted and time-limited, aligned with the principles of antimicrobial stewardship.

#### 2.4.3. Initiation of HCHA Pads

HCHA pads were initiated only after adequate wound bed preparation, including removal of devitalized tissue, control of local infection or critical colonization, and establishment of a granulation bed suitable for hydration-controlled healing. Their use was reserved for wounds demonstrating sufficient vascularized granulation tissue and requiring moisture modulation to support further tissue regeneration. Transition from the granulation phase to the epithelialization phase was guided by the development of a stable granulation bed, reduction of wound depth, and initiation of epithelial migration. HCHA pads were discontinued once moisture control was no longer required or when epithelialization progressed sufficiently to allow transition to dressings optimized for surface protection and moisture balance.

### 2.5. Multimodal Wound Care Decision Framework

Management of chronic wounds was conducted through a multimodal, patient-centered decision framework that integrated general supportive measures with targeted interventions selected according to predefined clinical criteria. Although treatment was individualized, the therapeutic choices followed a structured algorithm based on wound characteristics and patient comorbidities, reflecting real-world practice in an advanced wound care setting. The decision-making process for individualized wound management involved the following steps:

- Assess patient factors → including general status, comorbidities, and metabolic needs.
- Initial wound assessment → to classify healing phase (detersion/granulation/epithelialization).
- Evaluate infection risk → considering bacterial overgrowth/superinfection.
- Select the appropriate dressing type according to wound evolution → this included basic cleansing, absorbent dressings, collagenase for debridement, moisture-balancing dressings for granulation, and epithelialization-supporting dressings with HCHA pads.

**Figure 3:**
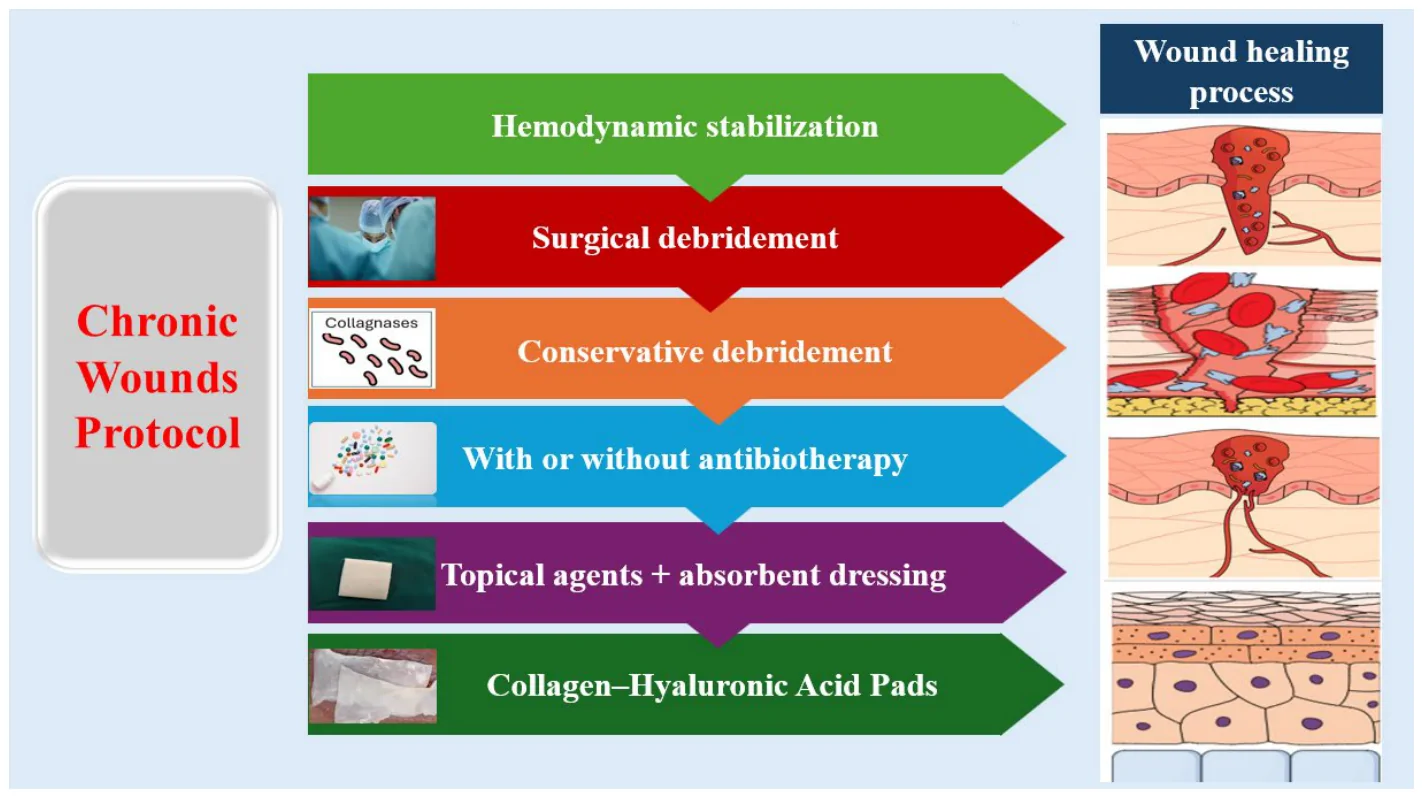
Multimodal therapeutic approach applied in chronic wounds of various etiologies, comprising excisional and conservative debridement with collagenase, targeted antibiotic therapy, and the use of topical agents and advanced absorbent hydro-active dressings to maintain optimal moisture balance.

Antimicrobial dressings or topical agents were introduced when clinical signs suggested bacterial burden, ensuring adequate infection control without delaying healing progression. This individualized approach, guided by a predefined algorithm based on wound characteristics and patient comorbidities, was chosen to reflect real-world clinical practice and to observe the adaptability and efficacy of HCHA pads within a patient-centered management strategy (Figure 3).

#### 2.5.1. Detersion Phase

In the debridement phase, wound bed preparation included excisional or conservative removal of devitalized tissue, supported when appropriate by enzymatic debridement with collagenase to facilitate biofilm disruption and restore a viable tissue surface.

#### 2.5.2. Granulation Phase

During the granulation phase, treatment focused on promoting vascularization and moisture balance using advanced absorbent or hydroactive dressings, selected based on exudate level and tissue quality.

#### 2.5.3. Epithelialization Phase

In the epithelialization phase, therapy prioritized protection of the fragile neoepithelium and stimulation of tissue regeneration, frequently employing HCHA pads to support matrix remodeling, until stable epithelial coverage was achieved (Table 1).

For wound disinfection, antiseptic agents are selected and applied based on the wound etiology, bacterial burden, tissue viability, and the presence of critical colonization or infection, to ensure an optimal balance between antimicrobial efficacy and tissue compatibility [30].

All patients underwent surgical debridement upon admission, performed under local anesthesia (9 cases), loco-regional anesthesia (24 cases), intravenous anesthesia (8 cases), or general anesthesia (23 cases). Following excisional debridement, collagenase was applied at 24–48 h and subsequently administered 3–7 times until complete wound bed cleansing was achieved.

To facilitate wound debridement, 42 patients (including 21 with burn injuries) were treated with hydro-active dressings containing superabsorbent polymers (SAPs) and Ringer’s solution, an advanced approach suitable for low-exudate wounds that provides continuous irrigation for 48–72 h. Multilayer high-absorption dressings were applied to 22 patients with high exudate.

For biofilm removal, 22 patients received multilayer high-absorption pads (≥120 g/100 cm^2^) incorporating a hydrophobic layer effective against bacteria, fungi, and endotoxins, enabling biofilm disruption without topical antibiotics and supporting healing without secondary risks.

Silver-based topical agents were used in 54 cases for antibacterial protection in various formulations. For superinfected wounds, topical agents containing silver ions were used as a powder spray in 14 patients with burns and 9 with chronic wounds, and as a cream in 18 patients with burns and 13 with chronic wounds. For exudative wounds, such as trophic ulcers, diabetic foot ulcers, and soft-tissue defects after tumor excision, we applied a powder spray containing a suspension of silver ions in hyaluronic acid.

For all wounds prone to dehydration and deepening, we used topical ointments containing hyaluronic acid combined with either collagenase (extracted from *Vibrio alginolyticus*) for gradual enzymatic debridement or silver sulfadiazine for localized antibacterial action. Low-molecular-weight hyaluronic acid (LMW-HA, 200 kDa) was applied to promote epithelialization and support scar remodeling.

**Figure 4:**
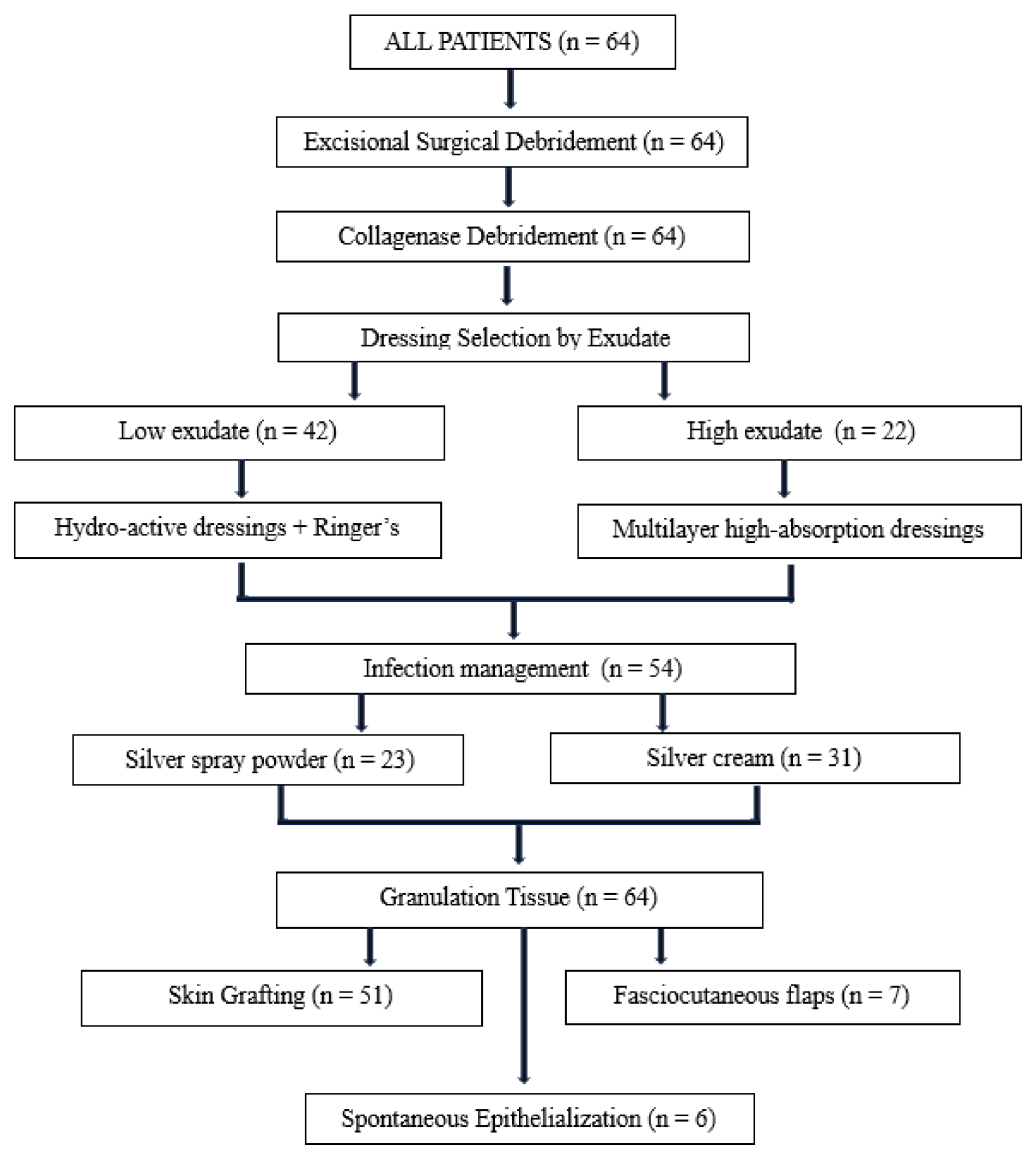
Schematic overview of the individualized multimodal wound management approach, illustrating the sequence of excisional and enzymatic debridement, dressing selection based on exudate level, infection control with silver-based topicals, and progression to HCHA therapy until granulation and wound closure.

We applied HCHA pads to all patients after surgical or conservative debridement of the wounds and used hydro-active dressings with superabsorbent polymers (SAPs), multilayer high-absorption dressings (≥120 g/100 cm^2^), or hyaluronic acid–silver spray powder, depending on the level of exudate and bacterial burden (Figure 4).

## 3. Results

The DHR ranged from 3.22% in burn wounds to 2.03% in non-burn chronic wounds. The overall healing duration ranged from 31 to 67 days for burn injuries and 34 to 87 days for non-burn chronic wounds (Table 2).

Despite involving larger skin areas, burn injuries demonstrated a faster healing rate, likely due to their predominantly superficial depth and intense pain that prompted earlier medical evaluation and treatment (Table S1). The DHR showed a narrow distribution across the cohort with burn injuries (mean 3.22%, SD 0.23%, 95% CI 3.14–3.30), indicating a consistent healing pattern among chronic burn wounds. A one-way ANOVA revealed statistically significant differences according to anatomical location (*p* = 0.014), with upper limb burns exhibiting the highest DHR (3.43%) and lower limb burns the lowest (3.07%). These findings align with the known regional variations in perfusion and mechanical stress.

Percent Area Reduction (PAR) was derived from the DHR using an exponential decay model, according to the formula:

PAR(t) = 1 − (1 − DHR/100)^t^,

where *t* represents the number of days of follow-up.

At 30 days, the PAR showed the greatest improvement in burns located on the upper limbs (mean PAR 67.2%), followed by burns involving multiple locations (64.7%) and trunk burns (63.7%). Lower-limb burns demonstrated the lowest PAR values (62.5%), although all anatomical regions showed substantial wound area reduction over the 30-day interval (Table 3).

Chronic wounds of non-burn etiology were more frequently associated with patients presenting multiple comorbidities, including vascular and neurological disorders, immunosuppression, or malignancy, which are factors known to impair tissue repair and substantially reduce healing capacity (Table S2). The Daily Healing Rate in the non-burn chronic wound cohort showed a narrow distribution (mean 2.04%, SD 0.20%, 95% CI 1.97–2.11), indicating consistent healing dynamics across patients. The one-way ANOVA revealed statistically significant differences among the etiologies (*p* = 0.0004). Postradiation and arterial ulcers demonstrated the lowest healing rates (1.68% and 1.73%, respectively), whereas complicated postoperative wounds and venous ulcers exhibited the highest DHR values (2.13% and 2.10%, respectively). These findings reflect the known variations in tissue perfusion, chronic inflammation, and local biological responses across different wound types (Table 4).

PAR in chronic wounds is widely recognized as a reliable marker for predicting overall wound-healing outcomes, especially in diabetic foot ulcers (DFUs), venous leg ulcers (VLUs), and other hard-to-heal wounds. PAR increased consistently over time in both burn and non-burn chronic wounds, with burn wounds demonstrating a markedly faster healing trajectory across all intervals. At 7 days, burn wounds showed a PAR of 20.0% compared with 13.2% in non-burn wounds. This difference widened at 14 days (36.0% vs. 24.6%) and was most pronounced at 30 days, with burn wounds showing a 62.0% reduction in wound area and non-burn chronic wounds a 45.2% reduction. These findings indicate a substantially accelerated healing dynamic in burn-related chronic wounds throughout the 30-day follow-up period (Table 5).

**Figure 5:**
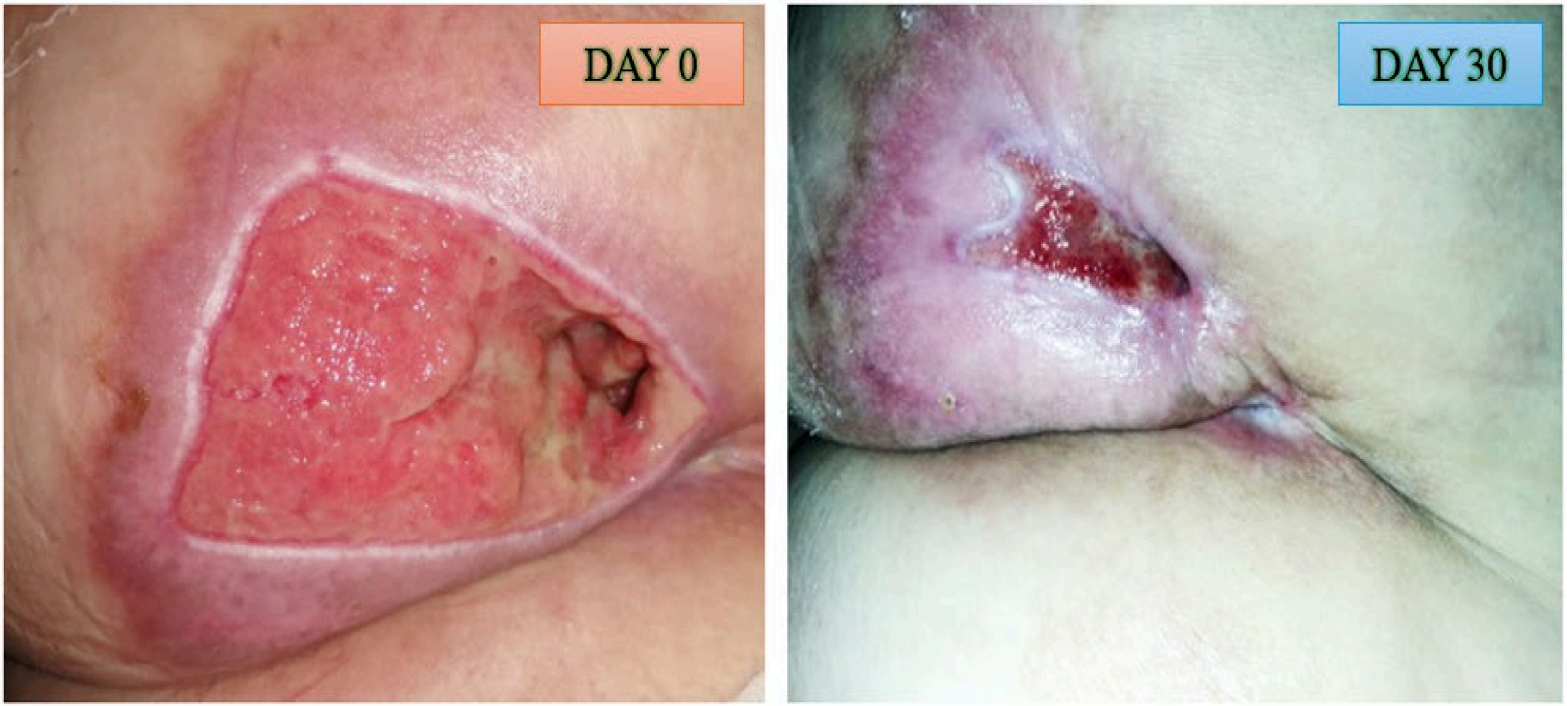
Stage III left gluteal pressure injury in a paraplegic patient after ischemic stroke. Following repeated debridement, a residual defect persisted without bone exposure or signs of osteolysis. The treatment included antiseptic cleansing, hyaluronic acid–silver spray, and HCHA pads. Images taken on days 0 and 30 showed progressive granulation and epithelialization.

Chronic wounds remain a major therapeutic challenge owing to delayed healing, persistent inflammation, and recurrent bacterial colonization. Advanced wound care strategies that integrate biomaterials with mechanical stimulation may enhance tissue regeneration and improve clinical outcomes. Figure 5 illustrates a representative case of a stage III left gluteal pressure injury in a paraplegic patient following an ischemic stroke. After repeated excisional and non-excisional debridement, a residual skin defect persisted without bone exposure, and no radiological or clinical evidence of osteolysis. Wound cultures remained negative throughout the follow-up period, and no systemic antibiotic therapy was required. The therapeutic approach included meticulous wound cleansing with antiseptic solutions, followed by the application of a hyaluronic acid–silver spray powder and HCHA pads, which were maintained in place for 48 h. Serial photographic documentation at baseline (day 0) and day 30 demonstrated progressive improvement in wound bed quality, with enhanced granulation and epithelialization.

**Figure 6:**
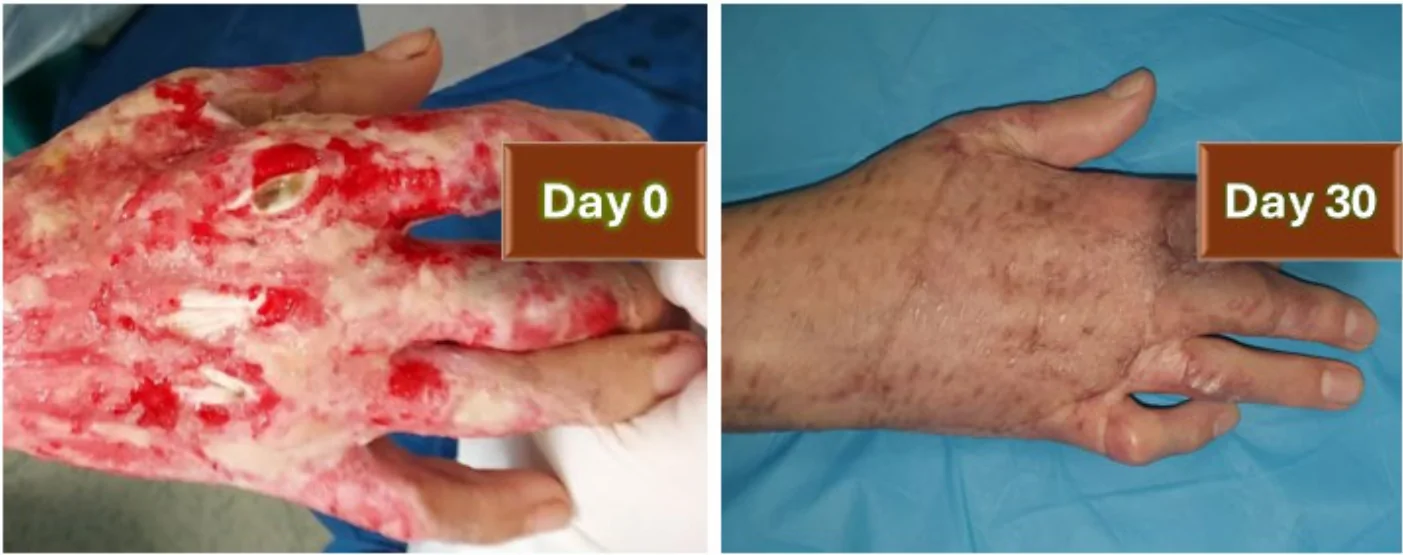
Second-to third-degree neglected burn injury located on the dorsal side of the right hand, with exposure of the extensor tendons after surgical and conservative debridement procedures.

The use of HCHA pads has been associated with favorable clinical progress in post-burn wounds with complex anatomical exposure (Figure 6). In the presented case, the dressing pads were applied to a dorsal hand burn complicated by exposure of the extensor tendons, a clinical scenario typically associated with delayed granulation, high risk of infection, and suboptimal functional outcomes. The HCHA pads was associated with a biologically favorable microenvironment that supported tissue formation and maintained adequate moisture balance, thereby protecting the exposed tendons from desiccation and secondary necrosis. A steady increase in epithelial coverage was documented over the course of treatment, with complete re-epithelialization achieved by day 30.

**Figure 7:**
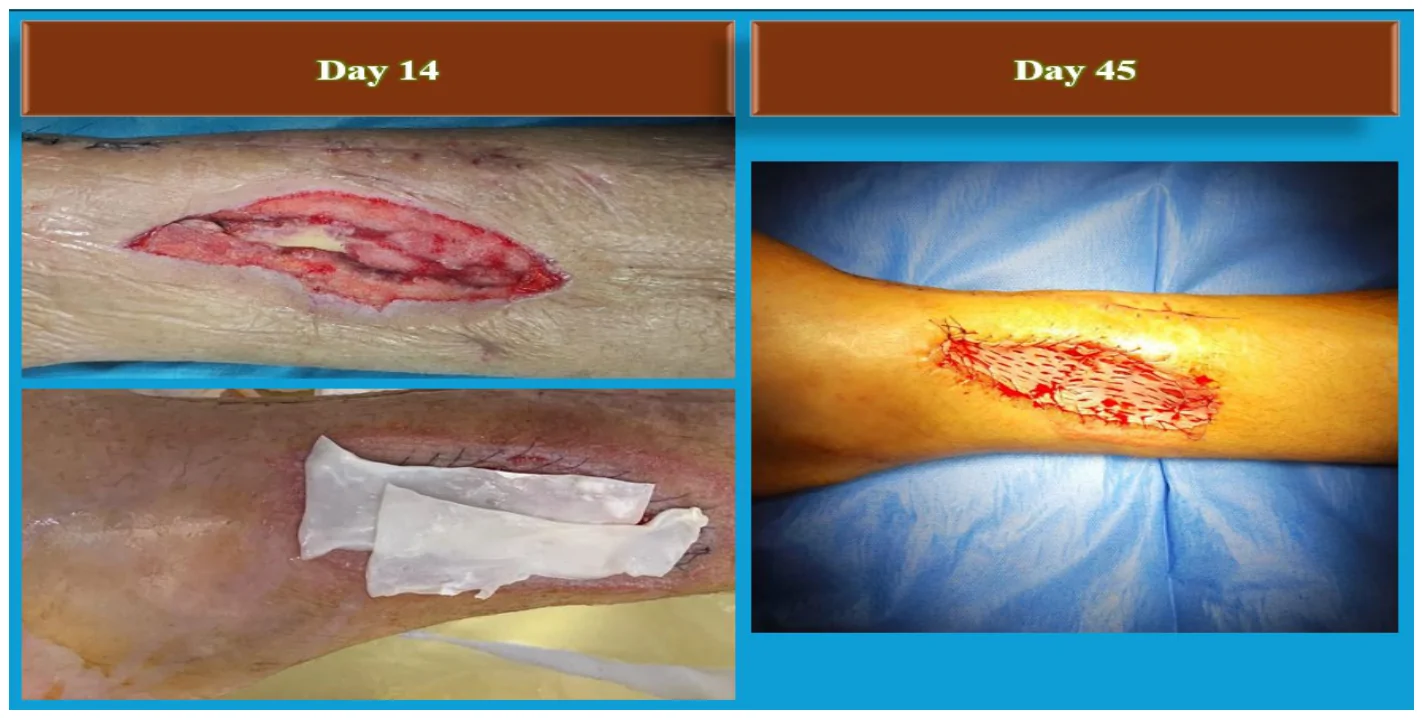
Soft-tissue defect on the medial distal lower leg following a crush injury with an open fracture of both lower leg bones. The application of HCHA pads was associated with granulation tissue formation, which covered the exposed tibia and allowed final reconstruction with a split-thickness skin graft.

In Figure 7, a soft-tissue defect is shown on the medial distal lower leg of a patient with a crush injury and an open fracture of both lower leg bones. Although the fracture was surgically stabilized, the trauma resulted in a residual defect with partial exposure of the tibia. HCHA pads were applied to protect the exposed bone and stimulate granulation tissue formation. Progressive granulation gradually covered the tibia and filled the defects. Once a stable, well-vascularized granulation bed was achieved, reconstruction was completed with a split-thickness skin graft, restoring durable coverage and supporting ongoing healing.

Procedural pain is an important, yet often under-recognized, component of chronic wound care, influencing treatment adherence and overall tolerability. To characterize changes in discomfort during wound cleansing and dressing changes, longitudinal Visual Analog Scale (VAS) scores were recorded for each patient throughout the treatment period (Table S3). This approach enabled a detailed evaluation of temporal nociceptive patterns, providing insights into the symptomatic burden and potential pain-modulating effects associated with HCHA pad use.

A comparative assessment of the two cohorts showed marked differences in both baseline pain intensity and the degree of improvement achieved throughout the treatment period (Table 6).

**Figure 8:**
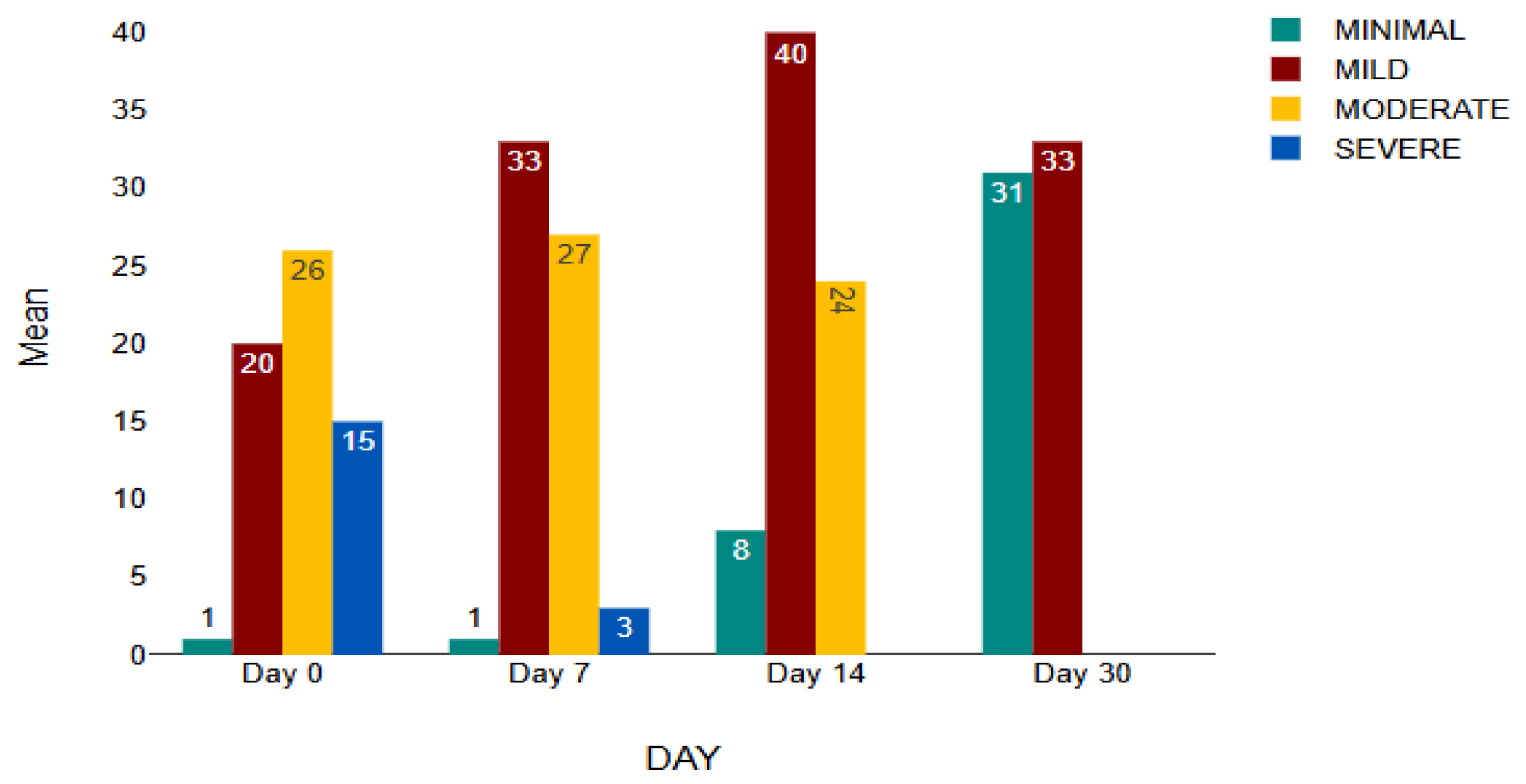
Longitudinal distribution of VAS pain scores from baseline to day 30.

Pain scores decreased significantly over time in both cohorts, but with distinct baseline levels and trajectories. Patients with neglected burn injuries reported higher initial pain (mean VAS 5.72; 95% CI: 5.14–6.30) than those with non-burn chronic wounds (mean VAS 4.53; 95% CI: 3.80–5.26). Despite this difference, both groups showed a consistent and statistically significant reduction in procedural pain at each follow-up interval (*p* < 0.001). By Day 30, VAS scores converged to similarly low levels—1.78 (95% CI: 1.52–2.04) in the neglected burn cohort and 1.53 (95% CI: 1.27–1.79) in the non-burn cohort. Effect sizes were large in both groups, indicating a strong clinical impact of the wound management protocol, with a greater magnitude observed in neglected burns (Cohen’s d = 2.47) than in non-burn chronic wounds (Cohen’s d = 1.94). These findings demonstrate that although burn wounds present with more severe initial pain, both wound types benefit substantially from the applied treatment strategy, achieving comparable pain reduction by day 30 (Figure 8).

### 3.1. Bacteriological Findings

Regarding the bacterial colonization/infection criteria, the initial evaluations showed the absence of microbial flora in 13 cases (20.31%), bacterial colonization of wounds in 21 cases (32.81%; saprophytic flora in 15 cases and pathogenic flora in six cases), and wound infection in 30 cases (46.87%). The main pathogens identified in infected wounds were Methicillin-resistant *Staphylococcus aureus* (MRSA) (12 cases), *Acinetobacter baumannii* (9 cases), *Pseudomonas aeruginosa* (4 cases), and mixed bacterial associations (5 cases). Serial bacteriological evaluations demonstrated that surgical debridement combined with antibacterial dressings was effective in 23 cases (76%), with clearance achieved within 10–30 days of treatment.

### 3.2. Clinical Outcomes Associated with Type I Collagen–Hyaluronic Acid Pads

Within the multimodal approach, HCHA pads were observed to protect anatomical structures and support granulation tissue formation.

- Five × five cm pads were preferentially used for tendon exposure on smaller, articulated areas such as fingers (flexor tendon zone 2: three cases; extensor tendon zones 2–3: four cases), toes (two cases), and Achilles tendon (one case), where precise coverage and flexibility were critical for protection and functional preservation.
- Ten × 10 cm pads were applied to wounds with bone exposure on the metacarpals and fingers (two cases), anterior tibial border (four cases), leg bones (three cases), and skull (two cases after tumor excision).

The dimensions of the smart dressings were tailored to the specific size of each wound.

### 3.3. Surgical Interventions

Patients underwent an average of two serial surgical debridements per case, with a range of:

- 3–5 debridements in burn wounds.
- 2–7 debridements in chronic wounds of other etiologies.

The highest number of debridements was required for diabetic foot lesions due to their complexity, presence of multiple purulent pockets, associated tenosynovitis or osteitis, polymicrobial flora with antibiotic resistance, and the need to preserve essential anatomical structures.

Soft tissue reconstruction was performed using:

- Split-thickness skin grafts in 46 cases.
- Full-thickness skin grafts in 5 cases.
- Fasciocutaneous flaps in 7 cases.
- Spontaneous epithelialization in 6 cases of small defects.

No adverse events related to the use of smart wound dressings, including Type I collagen–hyaluronic acid pads (HCHA), were documented throughout the study period. Clinical records did not report local intolerance, allergic reactions, increased exudation, wound deterioration, or any complications attributable to the dressings. All patients tolerated the materials well, and no treatment interruptions or modifications were required due to dressing-related issues.

## 4. Discussion

The management of chronic wounds remains a major clinical challenge because of the complex interplay among systemic comorbidities, local tissue conditions, bacterial colonization, and delayed healing. Early healing rates and wound area measurements have been extensively studied as predictive markers for complete healing of chronic wounds, including venous leg ulcers and diabetic foot ulcers [31]. The daily healing rate observed in our cohort highlights the important differences between wound types. Although burn injuries involve larger areas of the skin, they heal faster than chronic wounds of other etiologies. This finding is consistent with the pathophysiology of burn wounds, which often involve superficial tissue layers and prompt earlier medical intervention due to severe pain. In contrast, chronic wounds of non-burn origin typically occur in patients with multiple comorbidities, such as vascular disease, diabetes, depression, neuropathy, immunosuppression, or malignancy, which are well-known factors associated with impaired healing and prolonged recovery [32,33,34,35,36,37].

This study introduces an individualized, multimodal wound care approach, that integrates advanced dressings and tailored debridement strategies based on wound characteristics. This stepwise approach was adapted to the specific needs of each wound, beginning with appropriate debridement, followed by moisture-balancing or high-absorption dressings according to the exudate level, and transitioning to HCHA pads once an optimal wound bed was achieved. By structuring the therapeutic sequence in this manner, the approach accommodated variations in etiology, exudate burden, and tissue viability, ensuring that each wound received a personalized combination of interventions designed to support granulation and epithelialization.

The incorporation of HCHA pads was associated with the formation of a scaffold that mimics the natural ECM, facilitating cell attachment and new tissue formation. Studies on collagen-based hydrogels have demonstrated their ability to support fibroblast proliferation and vascularization, both of which are crucial for wound healing and tissue remodeling. Combining HA and collagen can stabilize the scaffold structure, improve cell viability, and enhance collagen expression in newly formed tissue [38,39]. The use of HCHA pads into an individualized multimodal approach introduces an additional option for managing chronic wounds.

By promoting angiogenesis, modulating inflammation, reducing oxidative stress, and enabling real-time monitoring of the wound microenvironment, these materials facilitate the development of a high-quality vascular bed, which is essential for tendon preservation. This optimized biological environment directly contributes to improved tendon gliding, reduced adhesion formation, and better long-term functional outcomes [25,40].

Conservative enzymatic debridement was used as indicated to optimize wound bed preparation before applying HCHA pads. This approach is consistent with current evidence supporting the role of advanced wound bed preparation strategies in facilitating subsequent granulation and epithelialization [41].

Overall, our findings reinforce the importance of a structured, multimodal approach to chronic wound management, in which effective wound bed preparation and bioactive dressing act synergistically. These findings align with existing literature suggesting that collagen dressings promote angiogenesis, support cell proliferation, and provide structural stability to the wound bed [42]. Beyond reinforcing these known mechanisms, our study provides specific insights into the comparative healing trajectories and pain reduction observed in a heterogeneous patient cohort receiving individualized multimodal care.

The differences observed between patient groups are consistent with the known biological effects of collagen–hyaluronic acid matrices on local inflammation. By attenuating excessive inflammatory signaling and improving extracellular matrix organization, the pads may enhance responsiveness to other components of the multimodal approach. This interaction likely explains the superior outcomes in patients with more pronounced inflammatory profiles at baseline.

### 4.1. Updated Comparison with Published Studies

Recent analyses confirm that Percentage Area Reduction (PAR) at 4 weeks is one of the most robust prognostic markers for subsequent wound healing. Thresholds around 50% PAR are consistently associated with a significantly higher probability of complete closure. In a large multicenter post-hoc analysis, Serena et al. (2024) [43] demonstrated that patients achieving PAR >50% at week 4 had a 65.4% probability of complete healing by week 12, whereas lower PAR values were strongly predictive of delayed or incomplete healing. These findings provide an important benchmark for interpreting real-world healing trajectories [43].

These PAR thresholds are used not only for prognostic purposes but also to compare the efficacy of different wound treatment modalities in clinical trials. In a recent systematic review, Lammert et al. (2024) [44] analyzed 23 clinical studies on hard-to-heal wounds and confirmed that early PAR thresholds—≥40% for venous leg ulcers and ≥50% for diabetic foot ulcers—demonstrate strong discriminative power in predicting complete healing. These validated cut-offs are increasingly adopted as surrogate endpoints in wound-care trials, allowing early comparison of treatment performance when complete closure is difficult to achieve within standard study durations [44].

Supporting this, Swain et al. (2024) [45] describe a case series employing a multimodal wound matrix (MWM) treatment that showed an average PAR of 57% after 4 weeks across diverse hard-to-heal wounds, including diabetic foot ulcers, venous leg ulcers, pressure injuries, postoperative wounds, and others. This PAR aligns well with the commonly observed PAR range and underscores that a 35–50% benchmark indicates a meaningful early wound-healing response in complex chronic wounds. This is likely attributable to their integrated design, which simultaneously addresses multiple barriers to healing—tissue non-viability, bioburden, moisture imbalance, and mechanical stress—while providing a bioactive extracellular matrix scaffold that supports granulation and early contraction. Such a multimodal strategy can produce accelerated early healing dynamics in appropriately selected patients, which may explain the relatively high PAR values reported in studies employing MWM protocols [45].

Additional interventional studies also support the relevance of early PAR thresholds. In a randomized controlled trial, Verdú-Soriano et al. (2023) [46] compared a novel hydrogel containing *Olea europaea* leaf extract (EHO-85) with standard hydrogel care in patients with chronic wounds. The authors reported a mean wound-area reduction of 51.6% in the EHO-85 group versus 18.9% in the control group over the treatment period, demonstrating that achieving or surpassing the ~50% PAR threshold is associated with markedly improved healing trajectories. These findings further reinforce the use of early PAR values as a meaningful comparative endpoint in clinical trials evaluating advanced wound therapies [46].

Moreover, pilot studies evaluating adjunctive therapies, such as blue-light photobiomodulation, have reported modest early healing responses. In a recent pilot trial, De Angelis et al. (2025) [47] observed a mean percentage area reduction of 40.5% at 12 weeks, with earlier time points showing non-significant trends toward area decrease. These findings illustrate the gradual nature of healing in chronic wounds and highlight the utility of PAR values in the 35–50% range as clinically meaningful milestones rather than absolute endpoints, particularly in studies involving hard-to-heal wounds or emerging therapeutic modalities [47].

Compared with these benchmarks, our findings show that burn wounds exhibited a distinctly more favorable early-healing trajectory. At 7 days, burn wounds already demonstrated a higher PAR than non-burn chronic wounds (20.0% vs. 13.2%). This divergence became most pronounced at 30 days, when burn wounds reached a 62.0% reduction in wound area—surpassing the 50% prognostic threshold reported in the literature—while non-burn chronic wounds remained below the level typically associated with accelerated healing. When stratified by etiology, our results show that arterial ulcers, postradiation ulcers, pressure sores, diabetic foot ulcers, and venous ulcers achieved mean DHR values between 1.68% and 2.13%, with PAR values of 39.8–49.4%. These outcomes are comparable to those reported in studies evaluating collagen–hyaluronic acid matrices, oxidized regenerated cellulose/collagen dressings, or hyaluronic acid–based wound therapies.

Collectively, these data demonstrate that PAR values in the range of 35–50% at early time points—particularly at 4 weeks—are frequently observed in the clinical management of hard-to-heal wounds and serve as valid surrogate indicators of healing trajectory. Consistent evidence from prognostic analyses, randomized trials, multimodal treatment case series, and pilot studies shows that wounds achieving PAR within or above this range are substantially more likely to progress toward closure. Consequently, such PAR thresholds provide clinicians with a practical, early means of evaluating treatment response and making informed prognostic judgments about wound-healing dynamics (Table 7).

By actively modulating the wound microenvironment rather than serving as passive barriers, these technologies address the key limitations of conventional dressings and support more efficient, individualized healing trajectories. These next-generation dressings integrate multifunctional biomaterials, sensing technologies, and programmable drug-delivery systems, which collectively enhance wound-care outcomes, especially in chronic wounds and diabetic foot ulcers [44,45,46,47,48,49,50].

Beyond the technical dimension, structured education enhances patient engagement, reduces anxiety associated with device use, and fosters a collaborative therapeutic environment that can significantly influence healing. These findings underscore the need to integrate patient-centered educational strategies into routine clinical practice, particularly when implementing complex or long-term wound care regimens [51,52].

While many studies focus on general healing rates, our explicit quantification of PAR in burn versus non-burn wounds contributes valuable data, especially given the recognized need for more specific PAR outcomes in the literature on advanced dressings [53,54].

### 4.2. Limitations

This study had several limitations:

- The study lacked a randomized or controlled design and individualized treatment protocols, and, while reflective of real-world practice, this means that direct comparisons between therapeutic modalities should be interpreted with caution, limiting the ability to isolate the precise effect of HCHA pads versus other protocol components.
- Treatment strategies were individualized rather than standardized, and although guided by predefined clinical criteria, the multimodal approach does not allow reconstruction of a uniform intervention protocol. This limits reproducibility and prevents clear attribution of outcomes to specific therapeutic elements.
- The study population was clinically heterogeneous, including burn wounds, diabetic foot ulcers, venous ulcers, arterial ulcers, pressure injuries, postoperative wounds, and post-radiation ulcers. These wound types differ substantially in pathophysiology, vascular supply, bacterial burden, inflammatory response, and healing kinetics. Although subgroup analyses were performed, this heterogeneity limits generalizability and complicates interpretation across etiologies.
- Confounding variables were not controlled through multivariable analysis, including baseline wound size, wound duration, infection status, vascular insufficiency, diabetic control, smoking status, nutritional status, BMI, and other comorbidities. Given the observational design, associations cannot be interpreted as causal.
- The use of laser Doppler imaging is limited to burn wounds, restricting the ability to compare vascularization dynamics across all wound types.
- Another limitation is the lack of long-term follow-up beyond complete wound closure. Post-healing outcomes, including scar maturation, hypertrophic or retractile scarring, functional sequelae, and recurrence, were not evaluated, and no therapeutic strategies for late complications were included. Consequently, the study reflects short-term clinical evolution rather than long-term reconstructive or functional outcomes.

### 4.3. Future Directions

- Future research should validate these findings in larger controlled clinical trials to better define the specific indications and patient populations that benefit most from Type I collagen–hyaluronic acid pads.
- Advanced imaging modalities, such as perfusion mapping and high-resolution ultrasound, may help clarify how biomaterial-enhanced therapies support granulation and protect exposed structures.
- Long-term follow-up studies are also needed to evaluate the functional outcomes, scar quality, recurrence rates, and patient-reported quality of life.
- In addition, the development of next-generation bioengineered dressings incorporating antimicrobial peptides, controlled-release growth factors, or customizable scaffold architectures offers promising opportunities to further enhance wound healing in complex clinical settings.

## 5. Conclusions

This retrospective observational study describes favorable clinical outcomes achieved within an individualized, multimodal wound care approach, that included HCHA pads. Beyond confirming progressive granulation and favorable healing trajectories, this study highlights a substantial reduction in procedural pain, thereby contributing to a more patient-centered therapeutic experience and suggesting a pathway to improve patient adherence and quality of life in chronic wound care.

The study also provides descriptive insight into the differential healing dynamics of burn and non-burn wounds treated within this multimodal framework. These observations suggest that HCHA pads may be a useful component for protecting vulnerable anatomical structures and supporting early tissue regeneration when integrated into a broader, individualized treatment strategy.

Although encouraging, these findings should be interpreted within the methodological constraints of the study. The retrospective design, absence of a control group, individualized treatment decisions, and clinical heterogeneity do not allow determination of the independent efficacy of HCHA pads. Instead, the results reflect real-world clinical evolution under an individualized multimodal approach and may serve as hypothesis-generating evidence for future controlled studies.

Smart wound dressings, including HCHA pads, represent a promising direction for further investigation. By improving wound bed conditions and promoting early tissue regeneration, these biomaterials show potential to reduce the need for complex reconstructive procedures, indicating a valuable direction for future investigation in managing challenging chronic wounds. Their potential to improve wound bed conditions, reduce procedural discomfort, and support early tissue regeneration warrants evaluation in prospective, standardized, and controlled clinical trials.

## Figures and Tables

**Table 1:** Algorithmic criteria for selecting debridement methods, dressing types, and HCHA pad application.

Clinical Parameter	Criteria	Intervention	Frequency/Duration	Number of Applications/ Patient
Healing phase: Detersion	Presence of devitalized tissue, fibrin, slough, biofilm	A single excisional debridement was performed for all patients, followed by enzymatic debridement with collagenase	Daily to every 48 h, depending on exudate and tissue response	3–7 applications/ patient
Exudate level: Low–Moderate	Moist wound bed; no maceration	Hydroactive or moisture-balancing dressings	Changed every 48–72 h	3–5 applications/ patient
Exudate level: Moderate–High	Risk of maceration; heavy drainage	Superabsorbent or multilayer absorbent dressings	Daily or every 48 h	3–8 applications/ patient
Infection risk	Signs of bacterial overgrowth or local superinfection	Adjunctive antimicrobial therapy (e.g., HA–silver spray powder/ointment)	Daily or every 48 h until resolution of signs	3–6 applications/ patient
Granulation phase	Healthy granulation tissue; moderate exudate	Hydroactive dressings + HCHA pads	HCHA every 48–72 h, depending on tissue response	4–6 applications/ patient
Epithelialization phase	Fragile neoepithelium; low exudate	Protective epithelialization-supporting dressings + HCHA pads	HCHA every 72 h until stable epithelial coverage	4–7 applications/ patient

**Table 2:** Daily Healing Rate (DHR) by Wound Category.

Wound Category	Number of Patients	Etiology Details	Initial Wound Surface (Reference)	Daily Healing Rate (DHR)	Total Healing Duration
Burn injuries	32	Grade 2B–3 burns; TBSA 7–10%; upper limbs (7), lower limbs (8), trunk (6), combined regions (11)	100% (baseline)	3.22%/day	The total healing duration ranged from 31 to 67 days.
Non-burn chronic wounds	32	Postoperative complicated wounds (14 cases) + trophic lesions (18 cases)	100% (baseline)	2.03%/day	Total healing duration ranged from 34 to 87 days

**Table 3:** Mean Daily Healing Rate (DHR) and Percent Area Reduction (PAR) at 30 days according to burn location.

Burn Location	*n*	Mean DHR (%)	Mean PAR (%)
Trunk	6	3.15	63.7
Upper limbs	6	3.43	67.2
Lower limbs	7	3.04	62.5
Multiple locations	13	3.22	64.7

**Table 4:** Mean Daily Healing Rate (DHR) and Percent Area Reduction (PAR) Across Non-Burn Chronic Wound Etiologies.

Etiology	*n*	Mean DHR (%)	Mean PAR (%)
Arterial ulcers	2	1.73	40.7
Pressure sores	4	2.04	45.9
Postradiation ulcers	2	1.68	39.8
Diabetic foot ulcers	5	2.00	45.7
Venous ulcers	5	2.10	48.3
Postoperative complicated wounds	14	2.13	49.4

**Table 5:** Percent area reduction (PAR) at 7, 14, and 30 days in burn and non-burn chronic wounds.

Interval	PAR in Burn Wounds	PAR in Non-Burn Chronic Wounds
7 days	20.0%	13.2%
14 days	36.0%	24.6%
30 days	62.0%	45.2%

**Table 6:** Longitudinal VAS Pain Scores during Wound Cleansing and Dressing Changes.

Group	Day	Minimal	Mild	Moderate	Severe
Non-burn wounds	Day 0	1	13	12	4
Non-burn wounds	Day 7	1	19	11	1
Non-burn wounds	Day 14	7	19	14	0
Non-burn wounds	Day 30	17	15	0	0
Burn wounds	Day 0	0	7	14	11
Burn wounds	Day 7	0	14	16	2
Burn wounds	Day 14	1	21	10	0
Burn wounds	Day 30	14	18	0	0

**Table 7:** This table summarizes Percentage Area Reduction (PAR) values from our cohort and key published studies. PAR reflects the proportion of wound surface reduction over early follow-up and serves as a validated surrogate marker of healing trajectory. Higher PAR values indicate more favorable early wound reduction and a stronger likelihood of progressing toward complete closure.

Study/Source	Study Type	Wound Types	Intervention Type	Reported PAR/Reduction %
Our study—Burn wounds	Retrospective observational	Burn wounds (trunk, limbs, multiple sites)	Type I collagen–hyaluronic acid pads	62.5–67.2% at 4 weeks
Our study—Non-burn wounds	Retrospective observational	Arterial, pressure, post-radiation, DFU, VLU, postoperative	Type I collagen–hyaluronic acid pads	39.8–49.4% at 4 weeks
Serena et al., 2024 [43]	Multicenter post-hoc	DFU, VLU, mixed chronic wounds	Standard of care dressings	>50% PAR at 4 weeks → 65.4% probability of healing at 12 weeks
Lammert et al., 2024 [44]	Systematic review	DFU, VLU	Various advanced dressings (collagen, ECM, hydrogels)	Validated thresholds: ≥40% (VLU), ≥50% (DFU)
Swain et al., 2024 [45]	Case series	Hard-to-heal wounds (DFU, VLU, pressure, postoperative)	Multimodal wound matrix (MWM)—ECM scaffold	57% PAR at 4 weeks
Verdú-Soriano et al., 2023 [46]	Randomized controlled trial	Chronic wounds	EHO-85 hydrogel (Olea europaea leaf extract) vs. standard hydrogel	51.6% (EHO-85) vs. 18.9% (control)
Angelis et al., 2025 [47]	Pilot study	Chronic wounds	Blue-light photobiomodulation device	40.5% PAR at 12 weeks

## Data Availability

The anonymized raw data supporting the findings of this study, including clinical parameters and patient outcomes, are available upon reasonable request from the corresponding author. To ensure patient privacy and confidentiality, access to raw data will be granted in accordance with institutional guidelines and data-sharing agreements.

## Supplementary Materials

The following supporting information can be downloaded at: https://www.mdpi.com/article/10.3390/jpm16080412/s1, Table S1. Distribution of Daily Healing Rate (DHR) in Chronic Burn Wounds (n = 32; mean = 3.22%). Table S2. Distribution of Daily Healing Rate (DHR) in Chronic Non-Burn Wounds (n = 32, mean = 2.03%/day). Table S3. Standard VAS Pain Categories. Table S4. VAS Pain Scores During Wound Cleansing and Dressing Changes. Cohort: 32 patients with neglected burn injuries. Table S5. Individual Longitudinal VAS Pain Scores During Wound Cleansing and Dressing Changes. Cohort: 32 patients with chronic non-burn wounds.
