# Supplementary material for: Clinical Outcomes of an Individualized Multimodal Chronic Wound Management Protocol Using Type I Collagen–Hyaluronic Acid Pads: Retrospective Observational Study
Source: J Pers Med. 2026 Jul 31;16(8):412. doi: 10.3390/jpm16080412 (PMC13514154; doi:10.3390/jpm16080412)
Supplement: Supplementary file 1 [file jpm-16-00412-s001.zip › jpm-4449079-supplementary.pdf]

Table S1. Distribution of Daily Healing Rate (DHR) in Chronic Burn Wounds (n = 32; mean = 3.22%).

**Pacient**	**Burn location**	**DHR (%)**
**1**	Burns located in the trunk	2.60
**2**	Burns located in the trunk	3.10
**3**	Burn injuries involving multiple locations	3.40
**4**	Burns located in the lower limbs	3.00
**5**	Burns located in the upper limbs	3.30
**6**	Burns located in the lower limbs	3.20
**7**	Burns located in the upper limbs	3.60
**8**	Burns located in the lower limbs	2.80
**9**	Burn injuries involving multiple locations	3.30
**10**	Burns located in the lower limbs	3.10
**11**	Burns located in the upper limbs	3.50
**12**	Burns located in the trunk	3.40
**13**	Burn injuries involving multiple locations	3.10
**14**	Burn injuries involving multiple locations	3.20
**15**	Burns located in the upper limbs	3.30
**16**	Burn injuries involving multiple locations	3.20
**17**	Burns located in the lower limbs	3.10
**18**	Burns located in the upper limbs	3.50
**19**	Burn injuries involving multiple locations	3.30
**20**	Burns located in the lower limbs	3.10
**21**	Burn injuries involving multiple locations	3.20
**22**	Burns located in the trunk	3.30
**23**	Burns located in the trunk	3.20
**24**	Burns located in the lower limbs	3.10
**25**	Burns located in the upper limbs	3.40
**26**	Burns located in the trunk	3.30
**27**	Burn injuries involving multiple locations	3.00
**28**	Burn injuries involving multiple locations	3.20
**29**	Burns located in the upper limbs	3.40
**30**	Burn injuries involving multiple locations	3.30
**31**	Burns located in the lower limbs	3.20
**32**	Burn injuries involving multiple locations	3.50

Table S2. Distribution of Daily Healing Rate (DHR) in Chronic Non-Burn Wounds (n = 32, mean = 2.03%/day).

Pacient	Chronic wound etiology	DHR (%)
1	Arterial ulcer	1.70
2	Pressure sores	1.80
3	Postradiation ulcer	1.85
4	Postoperative complicated wound - sacral region	1.90
5	Arterial ulcer	1.75
6	Postoperative complicated wound - scalp	2.00
7	Diabetic foot ulcers	2.00

8	Postradiation ulcer	1.50
9	Diabetic foot ulcers	2.05
10	Diabetic foot ulcers	2.05
11	Diabetic foot ulcers	1.80
12	Venous ulcer	2.10
13	Diabetic foot ulcers	2.10
14	Pressure sores	2.15
15	Postoperative complicated wound - posterior trunk	2.15
16	Pressure sores	2.20
17	Postoperative complicated wound - abdominal wall	2.10
18	Venous ulcer	2.20
19	Postoperative complicated wound - posterior trunk	2.25
20	Postoperative complicated wound - lower limb	2.05
21	Postoperative complicated wound - upper limb	2.30
22	Postoperative complicated wound - abdominal wall	2.10
23	Venous ulcer	2.30
24	Postoperative complicated wound - lower limb	2.25
25	Postoperative complicated wound - abdominal wall	2.15
26	Postoperative complicated wound - scalp	2.40
27	Postoperative complicated wound - lower limb	2.05
28	Postoperative complicated wound - posterior trunk	2.00
29	Venous ulcer	1.90
30	Postoperative complicated wound -sacral region	2.00
31	Pressure sores	2.00
32	Venous ulcer	2.01

Table S3. Standard VAS Pain Categories.

Category	VAS Range	Definition
Minimal	0–1	Pain is barely perceptible; no interference with wound care procedures
Mild	2–3	Low-intensity pain; well-tolerated, does not affect cooperation
Moderate	4–6	Clear, bothersome pain; may require brief pauses or gentler manipulation
Severe	≥7	Intense pain; significant discomfort during wound cleansing or dressing changes

Table S4. VAS Pain Scores During Wound Cleansing and Dressing Changes. Cohort: 32 patients with neglected burn injuries.

Patient	Day 0	Day 7	Day 14	Day 30	Pain Category (Day 0)
1.	7	6	4	2	Severe
2.	6	5	3	2	Moderate
3.	3	3	2	2	Mild
4.	5	4	2	1	Moderate
5.	7	5	3	2	Severe
6.	4	3	2	1	Moderate
7.	6	4	3	2	Moderate

8.	9	7	5	3	Severe
9.	5	4	2	1	Moderate
10.	3	3	2	2	Mild
11.	7	5	3	1	Severe
12.	8	6	4	3	Severe
13.	4	2	2	1	Moderate
14.	5	3	1	1	Moderate
15.	6	4	2	1	Moderate
16.	3	3	3	2	Mild
17.	8	6	3	2	Severe
18.	5	4	2	1	Moderate
19.	6	4	3	2	Moderate
20	7	5	4	3	Severe
21.	4	2	2	1	Moderate
22.	5	3	2	1	Moderate
23.	6	4	3	1	Moderate
24.	8	6	4	2	Severe
25.	7	4	3	2	Severe
26.	5	3	2	1	Moderate
27.	3	3	2	2	Mild
28.	7	5	3	2	Severe
29.	3	3	2	1	Mild
30.	6	3	2	2	Moderate
31.	5	3	2	1	Moderate
32.	7	4	3	2	Severe

Table S5. Individual Longitudinal VAS Pain Scores During Wound Cleansing and Dressing Changes. Cohort: 32 patients with chronic non-burn wounds.

Patient	Day 0	Day 7	Day 14	Day 30	Pain Category (Day 0)
1.	5	2	1	1	Moderate
2.	3	2	2	1	Mild
3.	1	1	1	1	Minimal
4.	8	6	4	3	Severe
5.	5	3	2	1	Moderate
6.	3	3	2	1	Mild
7.	3	2	2	1	Mild
8.	4	3	3	2	Moderate
9.	7	5	4	3	Severe
10.	3	2	2	1	Mild
11.	8	6	5	3	Severe
12.	4	3	3	2	Moderate
13.	6	4	3	2	Modeate
14.	3	2	2	1	Mild
15.	2	2	1	1	Mild
16.	4	3	3	2	Moderate
17.	6	4	2	1	Moderate
18.	2	2	1	1	Mild
19.	3	2	1	1	Mild
20	3	2	2	1	Mild

21.	7	5	4	3	Severe
22.	4	3	3	2	Moderate
23.	3	3	2	1	Mild
24.	6	4	3	2	Mild
25.	5	4	3	2	Moderate
26.	8	7	4	3	Severe
27.	2	2	1	1	Mild
28.	4	3	3	2	Moderate
29.	3	3	2	1	Mild
30.	2	2	1	1	Mild
31.	7	6	5	3	Severe
32.	6	4	3	2	Moderate
